# Reliability of pelvimetry is affected by observer experience but not by breed and sex: A cross‐sectional study in beef cattle

**DOI:** 10.1111/rda.13814

**Published:** 2020-09-18

**Authors:** Johannes Vernooij, Florine de Munck, Evelien van Nieuwenhuizen, Edward Webb, Herman Jonker, Peter Vos, Dietmar Holm

**Affiliations:** ^1^ Department of Population Health Sciences Section Farm Animal Health Faculty of Veterinary Medicine Utrecht University Utrecht The Netherlands; ^2^ Department of Animal and Wildlife Sciences Faculty of Natural and Agricultural Sciences University of Pretoria Pretoria South Africa; ^3^ Department of Production Animal Studies Faculty of Veterinary Science University of Pretoria Onderstepoort South Africa

**Keywords:** cattle, experience, observer variability, pelvimetry, probit model

## Abstract

Pelvis size plays an important role to prevent dystocia in cattle caused by the foeto‐maternal disproportion in commonly primiparous females. The reproducibility and repeatability are two important aspects for the reliability of the measurements to use in the selection of cattle for culling. Pelvic measures were taken with a Rice pelvimeter from 224 young cattle (180 females and 44 males) of four beef breeds in South Africa. One experienced and two inexperienced observers each measured pelvic height and width twice. The proportion measurements with a maximum difference of 0.5 cm within animal compared with the first measurement by the experienced observer are around 80% and by the inexperienced observers around 50% for pelvic height and around 60% for pelvic width. Breed and sex do not affect the reliability of pelvimetry by an experienced observer. Under‐ and overestimation of pelvis size were observed in inexperienced observers, which seems to be unrelated to breed and sex.

## 1 INTRODUCTION

Dystocia in cattle has significant economic and welfare implications for the dam and her calf (Barrier et al., 2013; Mee, 2008). Dystocia occurs more commonly in primiparous females due to foeto‐maternal disproportion. In heifers, dystocia is associated primarily with the birthweight of the calf and secondarily with the intrapelvic dimensions of the dam (Hiew, Megahed, Townsend, Singleton, & Constable, 2016; Wolverton, Perkins, & Hoffsis, 1991). Nevertheless, the prediction of dystocia based on pelvic area is very poor: a significant proportion of heifers with high risk does not experience dystocia and vice versa (Anderson, Brinks, LeFever, & Odde, 1993; Holm, Webb, & Thompson, 2014; VanDonkersgoed, Ribble, Townsend, & Janzen, 1990). Wolverton et al. (1991) report the birthweight of the calf as the most important reason and maternal pelvis size of second importance to the occurrence of dystocia. In this sense, selection of breeding bulls for a low birthweight can influence the birth weight of the calves and therefore lower the risk of dystocia (Mee, 2008).

Selection of breeding cattle can be based on measurements of their pelvic dimensions. In the prediction of the risk for dystocia using intrapelvic dimensions, the levels of accuracy, repeatability and reproducibility of intrapelvic measurements are important. Van Donkersgoed, Ribble, Booker, McCartney, and Janzen (1993) classified the repeatability using three different within‐observer cut‐off values for Rice pelvimeter measurements as moderate, based on kappa values. Paputungan, Makarechian, and Liu (1993) found a statistically significant difference in mean intrapelvic height between experienced and inexperienced operators and consequently also in the intrapelvic area. They classified the repeatability of pelvimetry as moderate (0.53, 0.46 and 0.55 for height, width and area, respectively) based on the percentage of explained variance. The estimated operator variance was low compared with the residual variance, which indicates a limited contribution by different observers to the total variability. The study by Paputungan et al. (1993) concludes that although the Rice pelvimeter is an easy‐to‐use device the observer must be trained to take valid internal measurements of the pelvis.

Kolkman et al. (2009) classified the repeatability of carcass measurements and ante‐mortal pelvimetry with the Rice pelvimeter as good because the repeatability coefficient (Bland & Altman, 1999) for both methods is within the limits of agreement indicating other factors account for the lower agreement than both methods. The estimated Pearson's correlation coefficient between carcass measurements and pelvimetry measurements for intrapelvic height, width and area were moderate (0.56, 0.46 and 0.59 respectively). The estimated bias (mean difference) between measuring intrapelvic width by pelvimetry or measurement on the carcass was small (−0.2, 95% limits of agreement: −2.5 and 2.1 cm), but the bias was relatively larger for intrapelvic height (1.2 cm, 95% limits of agreement: −1.8 and 4.1) (Kolkman et al., 2009). Similarly, Hiew et al. (2016) showed a greater within‐observer and between‐observer coefficient of variation for intrapelvic height than for intrapelvic width. However in their study, intrapelvic width was greater than height, and 25% of animals had intrapelvic dimensions exceeding the scale of the Rice pelvimeter and were therefore assigned a maximum score.

To our knowledge, apart from the importance of the level of experience of observers, distinct reasons that explain the differences/variation of measuring intrapelvic dimensions have not been studied. Therefore, the aim of this study was to evaluate the influence of breed and sex taking the observer experience level into account on the differences between repeated intrapelvic measurements compared with a measurement of an expert.

## 2 MATERIAL AND METHODS

### 2.1 Animals and measurements

In a cross‐sectional study, data were collected between the 21 July and 5 August 2015, from six different farms in central South Africa: one farm with Brahman (*n*_1_ = 36), from two farms with Nguni (*n*_2,1_ = 37, *n*_2,2_ = 17), from two farms with Bonsmara (*n*_3,1_ = 35, *n*_3,2_ = 40) and one farm with Hereford (*n*_4_ = 59) cattle. On each farm, one group of all young nulliparous females and males were measured except for the farm with Hereford cattle with 2 separately managed animal groups. Animals were moved down a crush chute on a flat area where they were individually restrained in a neck clamp for morphometric and pelvimetric measurements. The following data were collected: date of birth, sex, pregnancy status in females, body length (cm) measured from the bony prominence of the shoulder joint (cranial point of the major tubercle of the humerus) to the bony prominence of the hip joint (trochanter major of the femur), height at the withers (cm) measured vertically from the level ground surface to the highest point of the withers and heart girth (cm) measured as the circumference of the chest at the narrowest point just caudal to the front limbs.

Intrapelvic dimensions (cm) were measured using the Rice pelvimeter in increments of 0.5 cm as previously described (Hiew et al., 2016; Holm et al., 2014). Specifically in this study, the intrapelvic height was measured by (a) palpating the symphysis pubis trans‐rectally and (b) guiding the one caliper of the Rice pelvimeter onto the symphysis pubis with the hand inside the rectum and then (c) opening the pelvimeter until the other calliper was stopped by the ventral surface of the sacrum, in the midline. The intrapelvic width was then measured by (a) pushing the pelvimeter beyond the shafts of the ilii, then (b) opening the pelvimeter to the maximum distance before (c) retracting the pelvimeter gently so that the shafts of the ilii closed the pelvimeter to the maximum distance between the two shafts. Three observers each measured the intrapelvic dimensions twice on the same day: once in the morning and once in the afternoon. The order of measurement by the observer was the same in all animals (observer 1, 2 and then 3). Observer 1 (DH) was a veterinarian experienced in using the Rice pelvimeter (>10 years) from the veterinary faculty of the University of Pretoria, South Africa. Observer 2 (EN) and 3 (FM) were Master students in veterinary science from Utrecht University, the Netherlands. The Master's students were trained in advance, initially in transrectal palpation and later including pelvimetry under the supervision of the experienced veterinarian in two‐hour sessions 3 to 4 times in one week before starting the study.

### 2.2 Data handling

Data were checked and if needed corrected after entering in MS Excel, followed by calculating intrapelvic area (cm^2^) by multiplying intrapelvic height (cm) and intrapelvic width (cm). In one first measurement of observer 2 (EN), the value for intrapelvic width was higher than for the height which was very implausible given the values of the other repeated measurements. Therefore, the values for height and width of this measurement were interchanged. The farms participated voluntarily after giving informed consent. The project was approved by the University of Pretoria Animal Ethics Committee project number V089‐13.

### 2.3 Statistics

The first measurement of DH was considered as the reference measure to be able to assess the similarity of a second measurement of the experienced observer as well as both measurements of the inexperienced observers. The most experienced observer and his measurements are considered to be the best estimates for the pelvis size. The reference measurement is used to calculate the differences with the other five measurements within an animal for pelvis height and width respectively. The calculated difference will result in a positive value when the measurement is larger (overestimation) than the reference value and a negative difference means underestimation. The increments in the original measurements are 0.5 cm consequently leading to the same increments for the differences with the reference measurements.

The differences were summarized in frequency tables and visualized in bar plots per observer (1, 2 and 3) and measurement number (1 and 2), breed and sex, respectively.

The size of difference (ordinal) in pelvis height and width, respectively, was analysed using an ordinal regression model with cumulative link function (probit regression) with animalID as a random effect to take the correlated observations into account (package brms; Bürkner, 2018). The assumption was made that the underlying variable for the difference is continuous. Differences in height and width ≤ −2 and ≥ +2, respectively, are recoded as such to overcome problems in the estimation. Pregnant and non‐pregnant females were combined in one category because pregnant females were not present in all breeds. A model for pelvis height and width, respectively, was applied for each observer separately with breed and sex as explanatory variables as the full model with observer, breed and sex including interaction terms did not run properly. The results of the full model are presented as estimates with credibility intervals on the probit scale. Analyses were applied in R version 3.6.0 (R Core Team, 2019).

## 3 RESULTS

### 3.1 Study population

The number of animals per sex and descriptive statistics of age, morphometric and intrapelvic measures within a breed are presented in Table 1. Pregnant heifers were only present in Bonsmara and Hereford groups. Nguni cattle had on average the smallest intrapelvic height, width and area, and Herford cattle have the largest pelvis. This was also observed in the three different frame sizes, body length, hearth girth and height at the withers.

**Table 1:** Descriptive statistics of sex, age, morphometric and pelvis measurements by breed in four South African beef breeds

	Bonsmara	Brahman	Hereford	Nguni
Non‐pregnant females (*n*)	39	25	29	37
Pregnant females (*n*)	30	0	20	0
Males (*n*)	6	11	10	17
Age (days) mean (SD)	841 (190)^b^	530 (175)	653 (158)	672 (63)
Range	581−1097^b^	236–887	206–864	568–871
Pelvic Height (cm)^a^ mean (sd)	15.6 (1.89)	15.1 (1.00)	15.7 (1.91)	14.1 (1.18)
Range	12–20	13–17.5	11–19	12–17
Pelvic Width (cm)^a^ mean (sd)	12.5 (1.65)	10.2 (1.36)	13.7 (2.48)	9.7 (0.97)
Range	9–16	8–13	7.5–17	7.5–12.5
Pelvic Area (cm)^a^ mean (sd)	197.2 (46.2)	154.1 (27.4)	217.7 (57.7)	138.2 (23.1)
Range	120–307.5	112–208	90–304	90–212.5
Body length (cm) mean (sd)	118.3 (8.6)	118.9 (8.1)	120.3 (10.0)	110.7 (7.7)
Range	102–141	102–140	98–135	98–131
Height at the withers (cm) mean (SD)	119.8 (6.2)	119.8 (6.8)	122.5 (6.7)	111.8 (7.1)
Range	107–132	108–134	109–137	101–127
Heart girth (cm) mean (sd)	170.5 (10.9)	165.1 (13.3)	190.1 (15.4)	146.9 (11.0)
Range	148–203	140–199	154–234	124–175

Abbreviations: cm = centimetre*, n* = number of animals, sd = standard deviation.

^a^ From first measurement of observer 1.

^b^ Birth dates were unknown on one farm.

### 3.2 Differences of repeated measurements with the measurement of an expert

The differences of repeated measurements vary between −3.5 and +3.0 cm though the majority of the differences are between −1.0 and +2.0 cm (≥93%). About 86% of the differences for observer 1 is between −0.5 and +0.5 cm, and for observers 2 and 3, this percentage varies between 43% and 56%. The distribution of the differences for the second measurement of the observer 1 has a small dispersion and seems symmetric around zero (no difference between both measurements) in contrast with the distributions of the differences of observers 2 and 3. The distribution for the inexperienced observers is shifted to positive differences meaning more often overestimation of the pelvis height compared with the first measurement of observer 1. For observer 1, the percentage overestimation (26%) is similar to the percentage of underestimation (28%). The percentage overestimation for observers 2 and 3 is between 60% and 74%, and underestimation is between 10% and 18%, respectively. The second measurement of observer 2 and 3 shows some improvement towards smaller differences (differences between ±0.5 cm increase with +3% and +9% for respective observers). Detailed information on the distribution of the differences in pelvic height are summarized per observer and measurement sequence and presented in Appendix (Table S1).

Also for pelvis width (details in Appendix Table S2), the differences are between −3.5 and 3.0 cm. About 76% of the second measurement of observer 1 is within −0.5 and +0.5 cm, and for the inexperienced observers, this varies between 56% and 63%. The percentage of overestimation and underestimation is similar for observer 1. The inexperienced observers overestimate more often the horizontal pelvis size compared with the results of observer 1. For observer 2 and 3, overestimation is, respectively, between 40% and 54% and underestimation between 22% and 31%. Differences of the two master students seem to be closer to the distribution of difference of the first observer with increasing experience, that is in second measurements (differences between ± 0.5 cm increase with +5% and +4% for respective observers).

**Figure 1:**
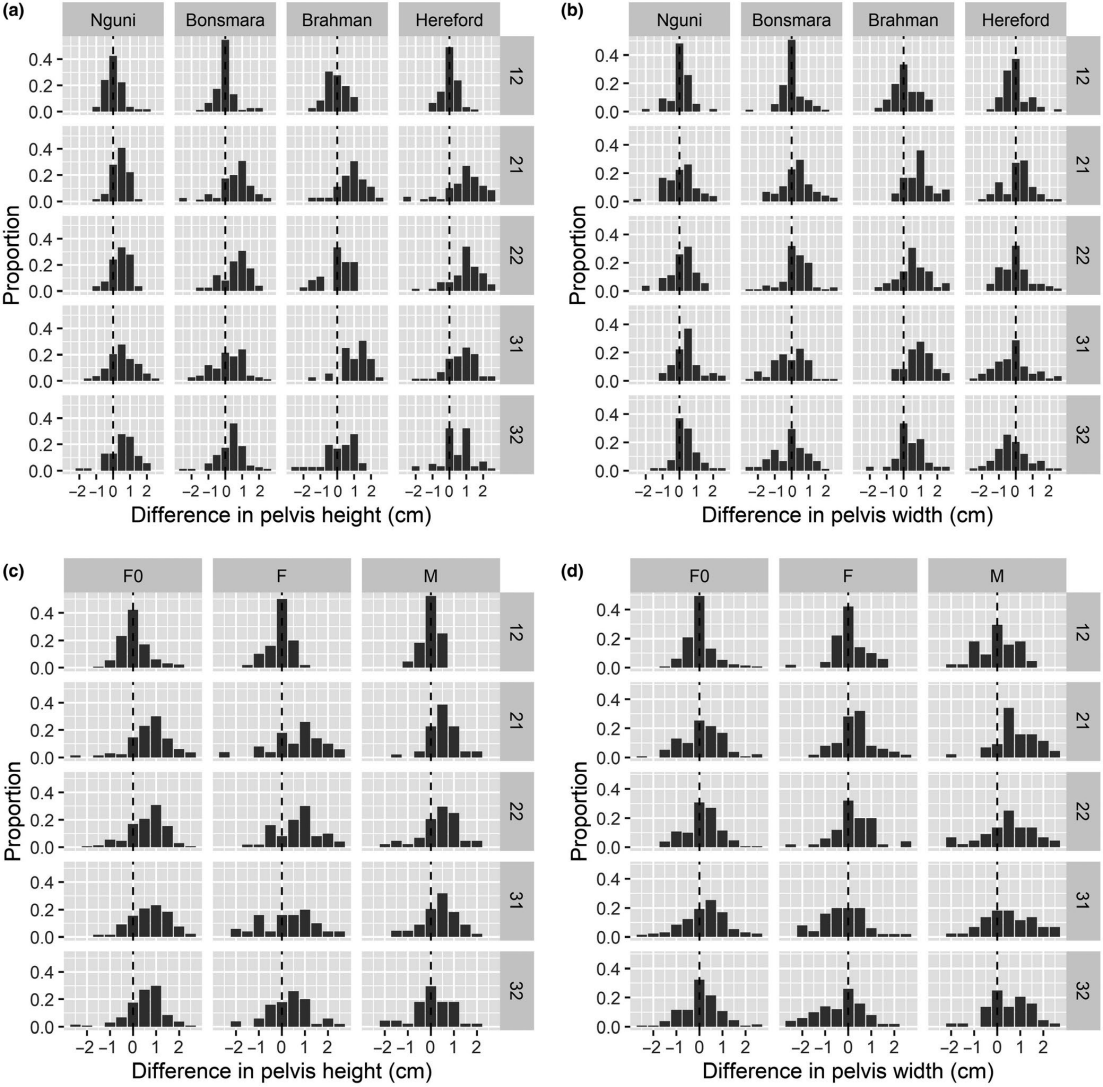
Plots of differences between repeated pelvis measurements and the first measurement of observer 1 (experienced) within an animal. The graphs present per observer and measurement (right side of graph), breed and sex (non‐pregnant females (F0), pregnant females (F) and males (M)) the proportion of differences in pelvic height (a and c and in pelvic width (b and d), respectively. Vertical dashed lines indicate no difference

A correct insight in the effect of breed and sex in the reliability of pelvimetry is difficult to obtain when all data are summarized without discriminating per observer (Figure 1). In Tables S3–S6 of the Appendix, the differences are summarized per breed and sex for observer 1. Figure 1 shows a clear pattern of distributions of differences shifted to overestimation by observer 2 and 3 within each breed (Figure 1a and b) and sex (Figure 1c and d), respectively. The second measurement of observer 1 is around 0 within each breed and sex. In Brahman cattle, the distribution of the difference in pelvis height and width seems to be wider compared with other breeds within this observer.

## 4 RESULTS

The results of the applied models are presented in Table 2. The estimates present the difference in the distribution of the respective breeds versus Nguni breed and males versus females. The parameter estimates for the second measure of observer 1 are all close to zero with 95% credibility intervals showing no systematic difference between breeds and sexes.

**Table 2:** Estimates of the ordinal regression analysis with cumulative link function for the difference of repeated pelvic height and width measurements, respectively, with the first measurement of observer 1 (experienced). The model was applied per each measurement separately with explanatory variables breed and sex. Credibility intervals marked in *italics* indicate over‐ or underestimation

Observer	Parameter^b^	Difference^a^ in Pelvis height				Difference^a^ in Pelvis width			
		Estimate^c^	Standard Error^c^	Lower^d^	Upper^d^	Estimate	Standard Error	Lower	Upper
M1.2^a^	Bonsmara	−0.13	0.19	−0.49	0.25	−0.07	0.19	−0.45	0.29
	Brahman	−0.24	0.23	−0.69	0.19	0.02	0.23	−0.42	0.46
	Hereford	−0.01	0.20	−0.39	0.38	−0.25	0.20	−0.65	0.15
	Male	−0.01	0.18	−0.36	0.34	−0.07	0.19	−0.43	0.30
M2.1^a^	Bonsmara	0.28	0.19	−0.10	0.66	0.44	0.19	*0.07*	*0.82*
	Brahman	0.67	0.23	*0.21*	*1.13*	1.02	0.23	*0.57*	*1.46*
	Hereford	0.74	0.20	*0.34*	*1.13*	0.14	0.20	−0.24	0.52
	Male	−0.29	0.18	−0.63	0.05	0.86	0.18	*0.50*	*1.21*
M2.2^a^	Bonsmara	0.31	0.19	−0.06	0.71	0.25	0.18	−0.10	0.61
	Brahman	−0.45	0.22	*−0.88*	*−0.02*	0.55	0.22	*0.13*	*0.99*
	Hereford	0.85	0.20	*0.45*	*1.24*	−0.16	0.20	−0.54	0.23
	Male	−0.18	0.18	−0.53	0.17	0.38	0.18	*0.01*	*0.75*
M3.1^a^	Bonsmara	−0.53	0.19	*−0.90*	*−0.15*	−0.56	0.20	*−0.93*	*−0.16*
	Brahman	0.72	0.23	*0.27*	*1.16*	0.59	0.23	*0.17*	*1.03*
	Hereford	0.10	0.19	−0.29	0.48	−0.65	0.20	*−1.04*	*−0.25*
	Male	−0.52	0.18	*−0.87*	*−0.18*	0.20	0.18	−0.15	0.56
M3.2^a^	Bonsmara	−0.43	0.19	*−0.82*	*−0.05*	−0.39	0.19	*−0.76*	*−0.01*
	Brahman	−0.47	0.22	*−0.90*	*−0.03*	0.06	0.22	−0.38	0.49
	Hereford	−0.19	0.20	−0.57	0.19	−0.60	0.20	*−0.98*	*−0.21*
	Male	−0.57	0.18	*−0.93*	*−0.22*	0.53	0.18	*0.17*	*0.88*

^a^ Difference between measurement *j* of observer *i* with first measurement of observer *1*.

^b^ Compared with distribution of the differences in Nguni cattle and females, respectively.

^c^ Mean and standard deviation of the posterior distribution on the probit scale.

^d^ 95% Credibility interval for posterior mean.

Breed and sex effects in measurements of inexperienced observers show variability in parameter estimates. In Bonsmara cattle, one and four parameters show over‐ and underestimation, respectively, compared with cattle of Nguni breed. Brahman cattle show overestimation in five parameter estimates and underestimation in two estimates. The distribution of measurement differences in Hereford cattle can be considered as more similar to Nguni cattle as they are twice overestimated and twice underestimated. Two parameter estimates for males out of four show underestimation in pelvis height, and three out of four parameter estimates for pelvis width in males show overestimation.

At first measurement, seven parameters in inexperienced observers show overestimation against four parameters underestimation and at second measurement underestimation occurs more frequently; six times underestimation versus four times overestimation, respectively. In pelvis height, four out of 10 parameter estimates show overestimation in contrast to seven out of 11 in pelvis width.

## 5 DISCUSSION

The results show no systematic effect of breed and sex on parameter estimates for differences in pelvis height and width, respectively, within an experienced observer. Inexperienced observers are more likely to over‐ or underestimate pelvis height and width. About half of the 21 significant parameter estimates indicate overestimation but there is no indication that breed or sex drives over‐ or underestimation of pelvis size. Over‐ and underestimation seems to be a random process in inexperienced observers and is dependent on measurement session. Overestimation occurs more often at first measurement and underestimation more frequently at second measurement. An explanation can be that the inexperienced observers might have been more exhausted at second measurement as this session was performed in the afternoon at the same day.

By using the first measurement of the experienced observer as a reference, we were able to evaluate the consistency of repeated measurements of an experienced observer and compare the distribution of differences with those of inexperienced observers. Moreover, the measurements of an experienced observer can be biased due to incorrectly placing the pelvimeter and rounding errors as the increments of the reading are in 0.5 cm. Using the first measurements for comparison, the calculated difference with repeated measurements are misleading when the first measurement is incorrect. When assumed that the measurement of an expert is accurate then the second measurement of the expert should on average not deviate from the first measurement which is indeed the case in this study. The reliability of the measurements of the first observer could have been evaluated when at least one measurement of a second experienced observer would have been involved in this study. Equal measurements were observed in 46% of the observations in pelvic height and 44% in pelvic width of the experienced observer. Taking rounding errors into account then, 86% of measurements in pelvis height are within −0.5 and +0.5 cm from the first measurement. For pelvis width, 77% differences are within this range. In the study of Kolkman et al. (2009), the assessed 95% limits of agreement for the bias between carcass and pelvis height were −1.8 to 4.1 and for pelvis width between −2.5 and +2.1. The results of our study show it rarely occurs for the experienced observer that the difference between first and second measurement is larger than 1 cm. In this sense, the measurements of an experienced observer are precise (low variability) although the true pelvic size is not known.

The two inexperienced observers were trained in advance of this study in three to four two‐hour sessions in one week. They probably improved their skill level during the study but due to the intertwining of breed with time it was impossible to assess the effect of an improved skill level.

The cattle in our study were young, not habituated to be restrained and be handled by people. Measurements were performed without analgesic treatment, and the cattle might have moved more during the measurement, which may have increased the measurement error in intrapelvic size. Nevertheless, the proportion of small within‐animal differences was high for the experienced observer.

According to Paputungan et al. (1993), the Rice pelvimeter is an easy to use device but as also shown in our study novice observers must be trained thoroughly to take valid internal measurements of the pelvis. The measurements should in a long‐term training period frequently be compared with the measurements of an experienced observer.

Although pelvis height and width are both continuous variables, it is not possible to make very precise measurements (increments of 0.5 cm) in vivo with the pelvimeter device. Therefore, it was not possible to analyse the data by standard linear regression methods with normality assumption. An ordinal regression method with a probit link was used to analyse the data as it is assumed that the underlying variables have a continuous scale.

Limitation of this study is the lack of the true pelvic size for comparison with all six measurements to obtain accurate differences, but this is inevitable when measurements are taken in young breeding cattle. Pregnant females were not available in all breeds making it unable to compare reliability with young non‐pregnant cattle. The limited number of farms and the intertwining of farm and breed impede estimating improvement in reliability by the inexperienced observers. The increment of the readings of the pelvimeter is 0.5 cm introducing extra variability due to rounding the values. Automatic readings could reduce the rounding errors, and regression methods based on a normal distribution might be applicable as resulting estimates are easier to interpret.

In conclusion, pelvimetry applied by an experienced observer is a reliable method to estimate pelvis size. The reliability of pelvimetry is not affected by breed and sex but the experience level of the observer does. To increase the reliability of pelvimetry measurements taken by novice, observers should be compared with measurements of an experienced observer in training sessions.

## CONFLICT OF INTEREST

None of the authors have any conflict of interest to declare.

## AUTHORSHIP STATEMENT

JV has designed the study, analysed the final data, prepared and finalized the manuscript. FM and EN have collected the data, preprocessed and checked the data and started first step in analysis, added parts of the Material and Methods and critically read the manuscript. FH, PV and EW have critically read and discussed the results and the manuscript. DH designed the study, collected the data and critically read and discussed results and manuscript.

## Supporting information

Appendix S1

## Data Availability

The data that support the findings of this study are available at Yoda, Utrecht University at https://doi.org/10.24416/UU01‐D8VRS7.
