# Supplementary material for: Reliability of pelvimetry is affected by observer experience but not by breed and sex: A cross‐sectional study in beef cattle
Source: Reprod Domest Anim. 2020 Sep 18;55(11):1592–8. doi: 10.1111/rda.13814 (PMC7756854; doi:10.1111/rda.13814)
Supplement: Supplementary file 1 — Appendix S1 [file RDA-55-1592-s001.docx]

Supplemental Information

Vertical and horizontal pelvis size was measured from cattle of 4 breeds from 5 farms. Measurements were taken by a veterinarian with more than 10 years of experience with Rice pelvimeter and two veterinary master students trained in pelvimetry in advance of the measurements. The measurements were taken in a morning and afternoon session on one day by each of the observers resulting in 6 vertical and 6 horizontal measurements of the pelvis.

The first measurement of the experienced observer was taken as the reference measurement and the difference with the other 5 measurements were calculated (measurement *i* – measurement *1*).

Differences are summarized in tables (S1 – S6).

Table S1. Distribution within observer and measurement number of within animal difference between five repeated pelvis height measurements and the first measurement of observer 1 (experienced) within animal.

|  | Observer 1 | | Observer 2 | | | | Observer 3 | | | |
| --- | --- | --- | --- | --- | --- | --- | --- | --- | --- | --- |
| Measurement | 2 | | 1 | | 2 | | 1 | | 2 | |
| Difference (cm)* | n | prop | n | prop | n | prop | n | prop | n | prop |
| -3.5 | 0 | 0.00 | 2 | 0.01 | 0 | 0.00 | 0 | 0.00 | 0 | 0.00 |
| -3.0 | 0 | 0.00 | 2 | 0.01 | 0 | 0.00 | 0 | 0.00 | 1 | 0.00 |
| -2.5 | 0 | 0.00 | 0 | 0.00 | 0 | 0.00 | 0 | 0.00 | 1 | 0.00 |
| -2.0 | 0 | 0.00 | 0 | 0.00 | 2 | 0.01 | 3 | 0.01 | 5 | 0.02 |
| -1.5 | 2 | 0.01 | 3 | 0.01 | 5 | 0.02 | 6 | 0.03 | 2 | 0.01 |
| -1.0 | 14 | 0.06 | 8 | 0.04 | 9 | 0.04 | 12 | 0.05 | 8 | 0.04 |
| -0.5 | 46 | 0.21 | 7 | 0.03 | 17 | 0.08 | 18 | 0.08 | 25 | 0.11 |
| 0.0 | 103 | 0.46 | 38 | 0.17 | 35 | 0.16 | 37 | 0.17 | 45 | 0.20 |
| 0.5 | 43 | 0.19 | 52 | 0.23 | 50 | 0.22 | 49 | 0.22 | 56 | 0.25 |
| 1.0 | 9 | 0.04 | 62 | 0.28 | 66 | 0.29 | 48 | 0.21 | 57 | 0.25 |
| 1.5 | 4 | 0.02 | 27 | 0.12 | 26 | 0.12 | 33 | 0.15 | 13 | 0.06 |
| 2.0 | 3 | 0.01 | 15 | 0.07 | 11 | 0.05 | 13 | 0.06 | 9 | 0.04 |
| 2.5 | 0 | 0.00 | 8 | 0.04 | 3 | 0.01 | 5 | 0.02 | 1 | 0.00 |
| 3.0 | 0 | 0.00 | 0 | 0.00 | 0 | 0.00 | 0 | 0.00 | 1 | 0.00 |

n = number, prop = proportion within observer and measurement, cm = centimeter

* difference = measurement – (first measurement of observer 1) within animal

Table S2. Distribution within observer and measurement number of within animal difference between five repeated pelvis width measurements and the first measurement of observer 1 (experienced) within animal.

|  | Observer 1 | | Observer 2 | | | | | Observer 3 | | | | | | |
| --- | --- | --- | --- | --- | --- | --- | --- | --- | --- | --- | --- | --- | --- | --- |
| Measurement | 2 | | 1 | | 2 | | | 1 | | | 2 | | |  |
| Difference  (cm)* | n | prop | n | prop | n | prop | n | | prop | n | | prop |  |  |
| -3.5 | 0 | 0.00 | 0 | 0.00 | 0 | 0.00 | 1 | | 0.00 | 0 | | 0.00 |  |  |
| -3.0 | 0 | 0.00 | 0 | 0.00 | 0 | 0.00 | 0 | | 0.00 | 1 | | 0.00 |  |  |
| -2.5 | 1 | 0.00 | 1 | 0.00 | 1 | 0.00 | 1 | | 0.00 | 2 | | 0.01 |  |  |
| -2.0 | 1 | 0.00 | 1 | 0.00 | 3 | 0.01 | 8 | | 0.04 | 5 | | 0.02 |  |  |
| -1.5 | 2 | 0.01 | 8 | 0.04 | 7 | 0.03 | 7 | | 0.03 | 11 | | 0.05 |  |  |
| -1.0 | 18 | 0.08 | 21 | 0.09 | 19 | 0.08 | 22 | | 0.10 | 22 | | 0.10 |  |  |
| -0.5 | 42 | 0.19 | 21 | 0.09 | 23 | 0.10 | 32 | | 0.14 | 27 | | 0.12 |  |  |
| 0.0 | 98 | 0.44 | 51 | 0.23 | 62 | 0.28 | 43 | | 0.19 | 66 | | 0.29 |  |  |
| 0.5 | 31 | 0.14 | 59 | 0.26 | 56 | 0.25 | 51 | | 0.23 | 42 | | 0.19 |  |  |
| 1.0 | 20 | 0.09 | 33 | 0.15 | 31 | 0.14 | 30 | | 0.13 | 27 | | 0.12 |  |  |
| 1.5 | 8 | 0.04 | 15 | 0.07 | 13 | 0.06 | 14 | | 0.06 | 14 | | 0.06 |  |  |
| 2.0 | 2 | 0.01 | 8 | 0.04 | 4 | 0.02 | 8 | | 0.04 | 4 | | 0.02 |  |  |
| 2.5 | 1 | 0.00 | 5 | 0.02 | 4 | 0.02 | 6 | | 0.03 | 1 | | 0.00 |  |  |
| 3.0 | 0 | 0.00 | 1 | 0.00 | 1 | 0.00 | 1 | | 0.00 | 2 | | 0.01 |  |  |

n = number, prop = proportion within observer and measurement, cm = centimeter

* difference = measurement – (first measurement of observer 1) within animal

Table S3. Distribution per breed of within animal differences between five repeated pelvis height measurements and the first measurement of observer 1 (experienced).

| Breed | Bonsmara | | Brahman | | Hereford | | Nguni | |
| --- | --- | --- | --- | --- | --- | --- | --- | --- |
| Difference  (cm)* | n | Prop | n | Prop | n | Prop | n | Prop |
| -2.5 | 3 | 0.01 | 1 | 0.01 | 2 | 0.01 | 0 | 0.00 |
| -2.0 | 3 | 0.01 | 2 | 0.01 | 4 | 0.01 | 1 | 0.00 |
| -1.5 | 7 | 0.02 | 7 | 0.04 | 2 | 0.01 | 2 | 0.01 |
| -1.0 | 24 | 0.06 | 9 | 0.05 | 11 | 0.04 | 7 | 0.03 |
| -0.5 | 40 | 0.11 | 21 | 0.12 | 20 | 0.07 | 32 | 0.12 |
| 0.0 | 89 | 0.24 | 32 | 0.18 | 68 | 0.23 | 69 | 0.26 |
| 0.5 | 83 | 0.22 | 38 | 0.21 | 47 | 0.16 | 82 | 0.30 |
| 1.0 | 79 | 0.21 | 39 | 0.22 | 72 | 0.24 | 52 | 0.19 |
| 1.5 | 30 | 0.08 | 19 | 0.11 | 37 | 0.13 | 17 | 0.06 |
| 2.0 | 13 | 0.03 | 10 | 0.06 | 21 | 0.07 | 7 | 0.03 |
| 2.5 | 4 | 0.01 | 2 | 0.01 | 11 | 0.04 | 1 | 0.00 |

n = number, prop = proportion, cm = centimeter

* difference = measurement – (first measurement of observer 1) within animal

Table S4. Distribution per breed of within animal differences between five repeated pelvis width measurements and the first measurement of observer 1 (experienced).

| Breed | Bonsmara | | Brahman | | Hereford | | Nguni | |
| --- | --- | --- | --- | --- | --- | --- | --- | --- |
| Difference  (cm)* | n | Prop | n | Prop | n | Prop | n | Prop |
| -2.5 | 5 | 0.01 | 0 | 0.00 | 2 | 0.01 | 1 | 0.00 |
| -2.0 | 8 | 0.02 | 1 | 0.01 | 6 | 0.02 | 3 | 0.01 |
| -1.5 | 16 | 0.04 | 2 | 0.01 | 16 | 0.05 | 1 | 0.00 |
| -1.0 | 32 | 0.09 | 6 | 0.03 | 41 | 0.14 | 23 | 0.09 |
| -0.5 | 46 | 0.12 | 18 | 0.10 | 53 | 0.18 | 28 | 0.10 |
| 0.0 | 112 | 0.30 | 38 | 0.21 | 86 | 0.29 | 84 | 0.31 |
| 0.5 | 78 | 0.21 | 37 | 0.21 | 43 | 0.15 | 81 | 0.30 |
| 1.0 | 50 | 0.13 | 42 | 0.23 | 21 | 0.07 | 28 | 0.10 |
| 1.5 | 16 | 0.04 | 21 | 0.12 | 16 | 0.05 | 11 | 0.04 |
| 2.0 | 7 | 0.02 | 7 | 0.04 | 5 | 0.02 | 7 | 0.03 |
| 2.5 | 5 | 0.01 | 8 | 0.04 | 6 | 0.02 | 3 | 0.01 |

n = number, prop = proportion, cm = centimeter

* difference = measurement – (first measurement of observer 1) within animal

Table S5. Distribution per sex of within animal differences between five repeated pelvis height measurements and the first measurement of observer 1 (experienced).

| Sex | Female pregnant | | Female non pregnant | | Males | |
| --- | --- | --- | --- | --- | --- | --- |
| Difference (cm)* | n | Prop | n | Prop | n | Prop |
| -2.5 | 2 | 0.01 | 4 | 0.01 | 0 | 0.00 |
| -2.0 | 5 | 0.02 | 2 | 0.00 | 3 | 0.01 |
| -1.5 | 4 | 0.02 | 7 | 0.01 | 7 | 0.03 |
| -1.0 | 21 | 0.08 | 24 | 0.04 | 6 | 0.03 |
| -0.5 | 28 | 0.11 | 60 | 0.09 | 25 | 0.11 |
| 0.0 | 55 | 0.22 | 139 | 0.21 | 64 | 0.29 |
| 0.5 | 46 | 0.18 | 141 | 0.22 | 63 | 0.29 |
| 1.0 | 49 | 0.20 | 156 | 0.24 | 37 | 0.17 |
| 1.5 | 17 | 0.07 | 77 | 0.12 | 9 | 0.04 |
| 2.0 | 15 | 0.06 | 30 | 0.05 | 6 | 0.03 |
| 2.5 | 8 | 0.03 | 10 | 0.02 | 0 | 0.00 |

n = number, prop = proportion, cm = centimeter

* difference = measurement – (first measurement of observer 1) within animal

Table S6. Distribution per sex of within animal differences between five repeated pelvis width measurements and the first measurement of observer 1 (experienced).

| Sex | Female pregnant | | Female non pregnant | | Males | |
| --- | --- | --- | --- | --- | --- | --- |
| Difference (cm)* | n | Prop | n | Prop | n | Prop |
| -2.5 | 4 | 0.02 | 4 | 0.01 | 0 | 0.00 |
| -2.0 | 7 | 0.03 | 4 | 0.01 | 7 | 0.03 |
| -1.5 | 9 | 0.04 | 22 | 0.03 | 4 | 0.02 |
| -1.0 | 24 | 0.10 | 65 | 0.10 | 13 | 0.06 |
| -0.5 | 38 | 0.15 | 84 | 0.13 | 23 | 0.10 |
| 0.0 | 74 | 0.30 | 204 | 0.31 | 42 | 0.19 |
| 0.5 | 51 | 0.20 | 141 | 0.22 | 47 | 0.21 |
| 1.0 | 26 | 0.10 | 80 | 0.12 | 35 | 0.16 |
| 1.5 | 9 | 0.04 | 27 | 0.04 | 28 | 0.13 |
| 2.0 | 4 | 0.02 | 9 | 0.01 | 13 | 0.06 |
| 2.5 | 4 | 0.02 | 10 | 0.02 | 8 | 0.04 |

n = number, prop = proportion, cm = centimeter

* difference = measurement – (first measurement of observer 1) within animal
